# ER Stress, UPR Activation and the Inflammatory Response to Viral Infection

**DOI:** 10.3390/v13050798

**Published:** 2021-04-29

**Authors:** Mara Cirone

**Affiliations:** 1 Department of Experimental Medicine, La Sapienza University of Rome, Viale Regina Elena 324, 00185 Rome, Italy; mara.cirone@uniroma1.it; 2 Laboratory Affiliated to Istituto Pasteur Italia-Fondazione Cenci Bolognetti, 00185 Rome, Italy

**Keywords:** viruses, PPRs, UPR, inflammation

## Abstract

The response to invading pathogens such as viruses is orchestrated by pattern recognition receptor (PRR) and unfolded protein response (UPR) signaling, which intersects and converges in the activation of proinflammatory pathways and the release of cytokines and chemokines that harness the immune system in the attempt to clear microbial infection. Despite this protective intent, the inflammatory response, particularly during viral infection, may be too intense or last for too long, whereby it becomes the cause of organ or systemic diseases itself. This suggests that a better understanding of the mechanisms that regulate this complex process is needed in order to achieve better control of the side effects that inflammation may cause while potentiating its protective role. The use of specific inhibitors of the UPR sensors or PRRs or the downstream pathways activated by their signaling could offer the opportunity to reach this goal and improve the outcome of inflammation-based diseases associated with viral infections.

## 1. Viruses, PPRs, and Inflammation

Viruses entering into target cells are promptly recognized by different classes of molecules identified as PRRs, which are mainly represented by the family of Toll-like receptors, nucleoside-binding oligomerization domain containing-like receptors (NLRs), C-type lectin receptors (CRLs), and the cGAS/STING pathway [1,2]. PRRs sense the pathogen components, called pathogen-associated molecular patterns (PAMPs), and trigger a signaling cascade, which after the recruitment of adapter molecules, culminates in the activation of transcription factors such as nuclear factor (NF) kB, activator protein [3] 1 and signal transducer and activator of transcription (STAT) 3, promoting the release of type I interferon (IFN) and proinflammatory cytokines such as IL-1β, IL-6, TNFα, and chemokines. Mitogen-activated protein kinases (MAPKs), and interferon regulatory factors (IRFs) may be also activated by PRR signaling and strongly contribute to cytokine release [4]. However, to balance inflammation, anti-inflammatory and immune-suppressive cytokines such as IL-10 are also produced downstream of PRRs. Epithelial cells and immune cells, particularly those present at the mucosal barriers, are equipped with several PRRs in order to prevent the potential insults that invading pathogens could induce. In addition, other immune cells are recruited from blood to the infected tissue by chemokines and other chemotactic factors, amplifying the inflammatory response. In some cases, inflammation may be so intense that it creates pathological consequences for the infected host and paradoxically leads to an impairment of immune function, exacerbating the damage that viral infection mediates [5].

## 2. Viruses, ER Stress, UPR Activation and Inflammation

In addition to PRR signaling, viral infection activates the unfolded protein response (UPR) [6]. This response is initiated by the activation of inositol-requiring enzyme 1 (IRE1) α, PKR-like endoplasmic reticulum kinase (PERK), and activating transcription factor (ATF) 6, the three main UPR sensors, which in unstressed conditions, bind to 78-kDa glucose-regulated protein (GRP78), also called BIP, which maintains them in an inactive state. The accumulation of unfolded or misfolded proteins into the ER, which causes ER stress, attracts BIP, detaching it from the three sensors that are activated [7]. Viral infection, especially during the replicative cycle, induces the production of a high quantity of viral proteins, which accumulate in the ER, overwhelming its folding capacity. This causes UPR activation, although viruses may trigger such responses independently of ER stress through the viral kinase PKR or by hijacking the ER membranes to accomplish their replicative cycle [8,9]. UPR can be considered an adaptive process, as it s. Indeed, UPR leads to the reduction of protein translation through the activation of the PERK–eukaryotic translation–initiation factor (eIF) 2 axis, increases ER chaperone transcription, mRNA degradation, and protein catabolism via endoplasmic-reticulum-associated protein degradation (ERAD) or macroautophagy through IRE1α [10]. Macroautophagy plays an essential role in relieving cells from stress; besides IRE1α, the other two UPR sensors PERK and ATF6 are also able to trigger this catabolic process [11]. However, macroautophagy or the selective forms of autophagy such as xenophagy are often dysregulated by viruses as a smart strategy to prevent them reaching the lysosomes, where they may be eliminated by the lysosomal proteases [12]. By reducing autophagy, viruses may exacerbate ER stress and this may shift the prosurvival function of UPR into cell death, an effect that can be advantageous for viruses if the infected cells belong to the immune system [13,14,15,16]. Interestingly, activated ER stress and UPR may contribute to the production of proinflammatory cytokines. The activation of IRE1α binds to TNF receptor-associated factor (TRAF) 2 and phosphorylates IkB, leading to the activation of NF-κB [17]. This transcription factor, which has a central role in the inflammatory response, can also be activated by ATF6 via AKT and by the PERK/eIF2α axis, which by inhibiting protein translation, reduces the expression of NF-κB inhibitor IkB. The c-Jun N-terminal kinase (JNK), p38 and extracellular signal-regulated kinase (ERK) 1/2, and mitogen-activated protein kinases (MAPKs) that are strongly involved in the control of cytokine release may also be activated by the three UPR sensors [18]. Indeed, UPR activation may occur independently of the presence of microrganisms, such as in the case of the ER stressor thapsigargin, which induces sterile inflammation [19]. The production of reactive oxygen species [20] generated during ER stress and the protein re-folding process contributes to the UPR-mediated activation of NF-κB and MAPKs. In addition, it has been reported that the UPR sensors may bind to genetic cytokine regulatory elements, directly affecting the production of inflammatory, antiviral, and immune-suppressive cytokines [21]. Importantly, sterile ER stress or single PRR stimulation induce a mild inflammatory response that requires both PRR ligation and ER stress to occur concomitantly to become intense inflammation. This is also because PRR and UPR signaling intersects at multiple levels and converges in the activation of the major proinflammatory transcription factors [22,23]. 

## 3. UPR and PRR Cross-Talk

Interestingly, viruses may activate UPR through PRR signaling independently of ER stress. For example, TLR2 and TLR4 signaling has been reported to activate IRE1α through TNF-receptor-associated factor (TRAF) 6 and nicotinammide adenina dinucleotide fosfato (NADPH) oxidase 2 (NOX2) [24]. NOX activation by viruses, together with the reduction of the antioxidant response, results in an increase of ROS that enhances the inflammatory response. However, the PERK sensor may also trigger the antioxidant response through the activation of nuclear factor erythroid 2-related factor (NRF) 2, balancing ROS levels [25]. Moreover, both TLR-signaling and the PERK sensor activate STAT3, which strongly contributes to the production of IL-6 and IL-10, cytokines that in turn keep STAT3 phosphorylated in a positive regulatory circuit [26]. From these evidences, it appears that UPR and PRR signaling intersects and cooperates in inducing the production of antiviral, proinflammatory, and anti-inflammatory cytokines, as well as ROS. The inflammatory response in the course of viral infection may be particularly strong, as it concomitantly affects both PRR and UPR signaling in either an independent or interconnected fashion. This suggests that to mitigate excessive inflammation and prevent the local or systemic destructive effects that may occur during viral infection, the selective inhibition and activation of UPR sensors or PRR could represent a potential strategy.

## 4. Possible Consequences of Excessive Inflammation in Response to Viral Infection

**Figure 1:**
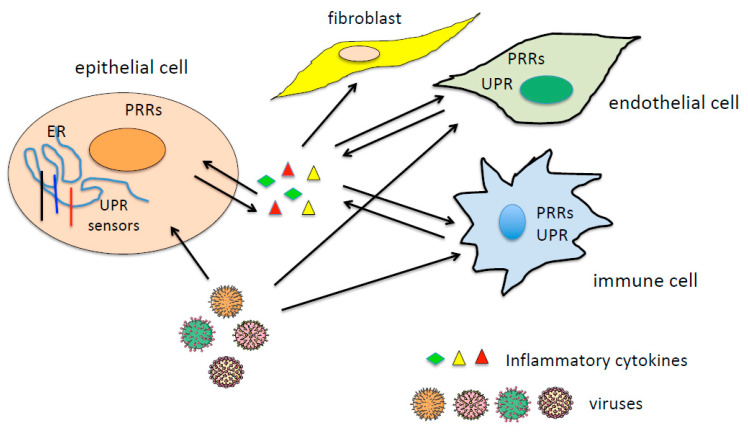
PRR and UPR signaling induce cytokine release that may spread the effects.

Some respiratory viruses, such as influenza [27] or coronaviruses [28], or herpesviruses, such as Epstein–Barr virus (EBV) or human cytomegalovirus (HCMV), when reactivating from latency in immune-compromised patients, infect epithelial cells, such as alveolar or bronchial cells, as well as endothelial and immune cells, leading to a massive proinflammatory cytokine release, known as “cytokine storm syndrome” [29]. This huge amount of cytokines can worsen the tissue damage induced by viral infection and alter the functions of uninfected bystander cells in the site of injury. Cytokine-damaged endothelial cells may result in activation of the coagulation cascade, given the strong interconnection that occurs between inflammation and the activation of the coagulation cascade [30]. The functions of fibroblasts may also be altered by the inflammatory milieu, as it stimulates their trans-differentiation into myofibroblasts, promoting fibrosis and thus aggravating tissue dysfunction [31]. The interplay between infected and uninfected cells mediated by the release of inflammatory cytokines is shown in Figure 1.

Finally, cytokines may be transported far from the site of production by the blood stream, causing damage in several organs and tissues and inducing systemic effects. These events are reported to occur in some patients following infection by the new coronavirus SARS-CoV2, which causes the pandemic disease known as COVID-19 [32]. 

## 5. Targeting UPR or PRR Signaling to Mitigate Excessive Inflammation in the Course of Viral Infections

Although the role of UPR activation in the release of proinflammatory, antiviral, and anti-inflammatory cytokines during viral infection is still under investigation, there are several studies showing that it contributes to inflammation [21,33]. The example of some viruses being able to activate UPR or one of its arms to enhance cytokine production is reported in Table 1. 

Therefore, to mitigate the destructive process of inflammation and improve the efficiency of the immune response, the selective inhibition of UPR sensors or PRR signaling could be exploited. In the first case, pharmacological inhibitors of IRE1α, PERK, and ATF6 molecules such as 4μ8c, Ceapin, or GSK2606414, respectively, could be used, although the side effects that they could cause must be considered [20,34]. Indeed, less toxic UPR inhibitors are being developed and some of them are in preclinical trials against cancers, such as multiple myeloma [3]. To control PRR signaling, a promising strategy could be to use the antagonists of TLR4, such as Eritoran. Targeting this particular TLR could be particularly efficient in restraining the destructive excess of inflammation, as TLR4, besides by PAMPs, it may be activated by molecules released following viral-induced tissue damage, called damage-associated molecular patterns (DAMPs) [35]. Therefore, it appears that tuning PRR or UPR signaling may help to reduce the severity of complications occurring in the course of viral diseases, particularly COVID-19 [36]. Regarding herpesviruses, the majority of which are ubiquitous viruses, they are known to infect and persist in several cell types, and the chronic infection that they cause may induce long lasting or chronic inflammation in several tissues. This facilitates the onset of a variety of inflammation-based diseases ranging from gastritis, inflammatory bowel diseases, autoimmune diseases, neurodegenerative diseases, and cancer [33,37,38,39]. Other completely different viruses such as hepatitis viruses may also cause diseases, to whose pathogenesis the inflammatory response strongly contributes. Therefore, a better understanding of the mechanisms regulating the intensity and duration of the inflammatory response could help to find strategies to selectively control the activation of PRRs and UPR signaling or their downstream activated pathways, restraining the side effects of inflammation while preserving or even potentiating its protective role, improving the outcome of virus-associated diseases.

**Table 1:** Example of viruses that promote inflammation by activating UPR.

Virus	UPR/UPR Arm	Inflammatory Molecules	References
Hepatitis B virus (HBV)	eIF2α/ATF4	Cox-2	[40]
Dengue virus (DENV)	PERK/Nrf2	TNF-α	[41]
Kaposi’s sarcoma-associated herpesvirus (KSHV)	IRE1a	TNF-α, IL-6, IL-10, IL-8, VEGF	[33]
	PERK	CCL-2	[33]
SARS-CoV-2	ER stress/UPR	IL-6, IL-1b	[36]

## Data Availability

Not applicable.
